# Are the People to Be Unknowingly Drugged?

**Published:** 1909-08

**Authors:** Paul Pierce

**Affiliations:** New York, N. Y., Editor the National Food Magazine


### Abstracts and Selections.

### Are the People to be Unknowingly Drugged?

BY PAUL PIERCE, NEW YORK, N. Y.,
Editor The National Food Magazine.

The average individual is usually careful in selecting his physician; and the average physician is safe to be skillful in selecting his drugs. But the same individual, without the exercise of unceasing care, is compelled unconsciously to take into his system daily many doses of drugs which have been incorporated into various more or less sanitarily prepared foods.

The idea of using embalming substances to preserve articles of daily diet is not attractive, although some commercial interests insist that they are necessary and harmless, and as this question of using chemical preservatives in food products is excitng such universal interest at the present time, there is a loud call for the opinion of the medical fraternity on the subject.

The reactionary food manufacturing interests are now clamoring for a return to the practice of using artificial preservatives without restraint of the fool control authorities. But there is another element of manufacturers who declare that chemicals are not necessary to preserve good food material, when perfect sanitation prevails. They class artificial preservatives as drugs, fit only for medicinal purposes, to be administered only by competent physicians, and it is the physician—the man who makes a study of disease and its cause—who can tell us more authoritatively than any one else, about the real effects of foods preserved with chemicals.

The general public probably does not note, but scientific people surely must have marked, the significance of the fact that, based upon the opinion of a Special Government Commission, appointed by the insistence of the class of manufacturing interests first referred to—the authorities at Washington have issued a ruling under the National Food Law, permitting the unlimited use of benzoate of soda, generally agreed, we believe, to be a dangerous coal-tar drug when taken for an indefinite period in prepared foods.

Employed in foods where it can not be detected by taste or smell, we learn that its principal use is to permit the cheap preservation of inferior raw materials and products carelessly prepared in indifferently clean surroundings.

Leaving out of consideration, however, the fact that the presence of benzoate of soda usually indicates the kind of food one would not care to eat who saw it made and knew what it was made of—the question of greatest importance, of course, is the possible injurious effect of this drug upon the health of the people. Under this point, there is admittedly a conflict of opinion among scientists, and the opinions against its wholesomeness are so numerous and come from sources so eminent as to constitute at least a doubt too grave in character, we think, to be ignored or lightly passed over.

It is a self-evident proposition that drugs are not foods, and should not be administered indiscriminately to sick and well, infant and adult, without the sanction of a physician.

To their everlasting credit, some food manufacturers stand strongly for this principle and have formed a powerful organization to fight for strict food purity and against the use of chemical preservatives, which they have proved by their own experience to be unnecessary, if fresh, clean materials are employed, under sanitary conditions.

Many reforms have been brought about.

Artificial colors have been driven out. Food labeling has been improved to make it more honest, but many abuses remain.

However, the eminent Commission, appointed, unfortunately, at the behest of the reactionary food manufacturers, to consider the harmfulness or otherwise of benzoate of soda, seems to have dealt with the matter from a chemical standpoint only, as was anticipated at the outset, and their decision has been taken advantage of to perpetuate some of the gravest abuses of the past.

Today, by the action of the government officials at Washington, that section of the National Food Law which aimed to secure freedom from adulteration and fraudulent cheapening, is practically nullified by the free admission into all foods of benzoate of soda as a preservative; and so far as the National Government is concerned, this drug may now be used in any quantity in meat, fish, milk, butter, cheese, vegetables, fruits and condimental foods; in fact, the entire list of our prepared food supplies.

It is admitted generally, and confirmed by Dr. Bitting of the Department of Agriculture, in Government Bulletin No. 119, of January 9, 1909, that the principal use of benzoate of soda is to permit the employment of refuse and waste material. This material is the garbage of the canning factory, more often than otherwise half rotten, sour and offensive at the outset; old, spoiled and vermin-infested evaporated apples and other fruits, preserved and manufactured into attractive-looking foods; and its manner of using in this way has been more fully and graphically described than it can be related here.

These are grave—not to say criminal—abuses, and the work of improving such conditions seems worthy of the attention of all public-spirited professional men.

Many of the leading pathologists and chemists of the country have demurred from the decision of the Government Commission, either on the ground that it was not warranted by any facts ascertained during the investigation, or by the fact that the inquiry was not broad enough to be practical, in that it failed to cover the usual purpose of artificial preservatives in foods, or both.

England, Germany, France, Italy, Austria and Spain all have food laws condemning and restricting chemical preservatives in food products. The World's Pure Food Congress, recently held at Geneva, Switzerland, condemned chemical preservatives. Dr. H. W. Wiley, Chief of the United States Bureau of Chemistry, has submitted official reports denouncing them as extremely injurious. Similar action has been taken by the Association of State and National Dairy and Food Departments.

As a rule, the press of the country has espoused the cause of the consumer as against that of the food adulterer and the corrupt politician, but no one not at the heart of the situation can realize the extent of the outrages perpetrated on the human stomach by certain types of food manufacturers.

The gravity of the situation demands expert and conscientious workers, and it is hoped that the medical fraternity of the country will seize upon this as labor worthy of their attention, and rise up and stamp out this menace to human health and normal living.

The writer has sent out some 6000 letters to prominent physicians, asking their opinions upon the use of chemicals as food preservatives, with regard to the health of the community. Already the answers are coming in, in large numbers, condemning in no uncertain terms the use of chemicals as unnatural and deleterious as articles of diet. Up to date, not one physician has written commending, or even condoning, the process.—*Lancet-Clinic.*

---

THE prognosis in tuberculous diseases of bones and joints in children has been improved more by the practical application of the fresh air treatment than by any other means. The next step in surgical enlightenment is to apply the same treatment to other surgical disease.—*American Journal of Surgery.*

